# Supplementary material for: A Non-Rogue Mutant Line Induced by ENU Mutagenesis in Paramutated Rogue Peas (Pisum sativum L.) Is Still Sensitive to the Rogue Paramutation
Source: Genes (Basel). 2021 Oct 23;12(11):1680. doi: 10.3390/genes12111680 (PMC8623080; doi:10.3390/genes12111680)
Supplement: Supplementary file 1 [file genes-12-01680-s001.zip › genes-1379240-supplementary.pdf]

## SUPPLEMENTARY INFORMATION:

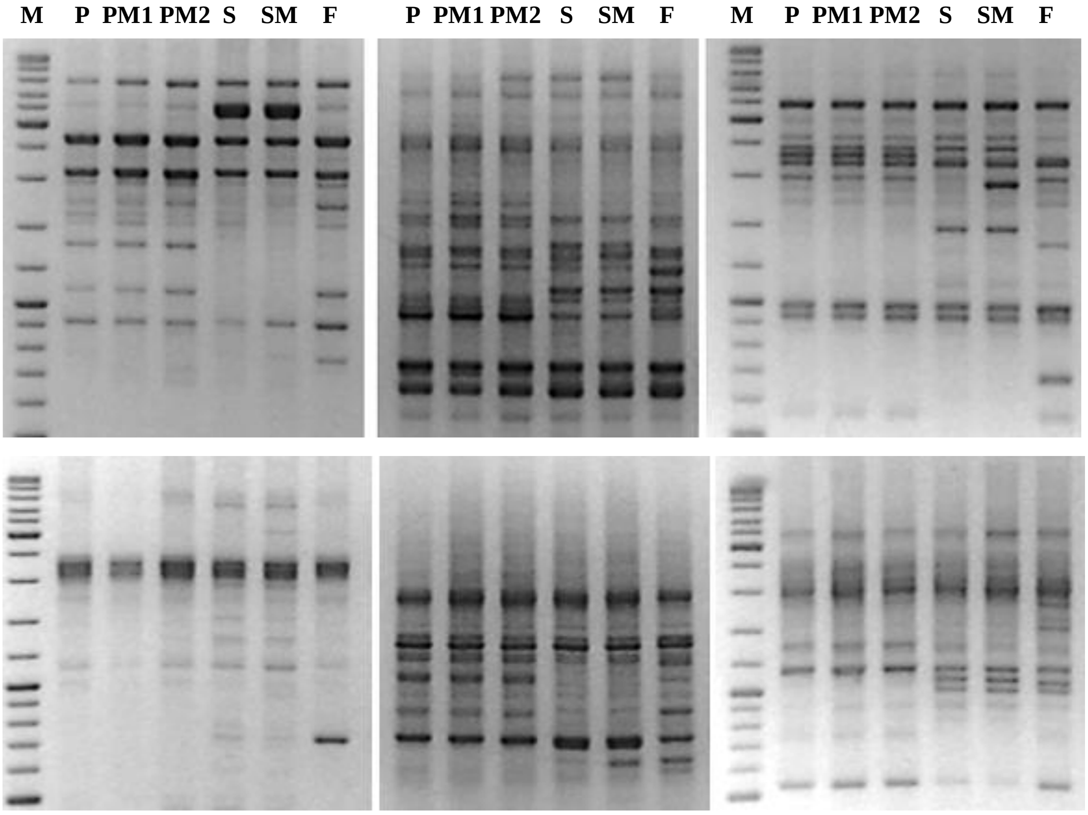


**Figure S1**. RAPD patterns of: **P -** cv. Progreta; **PM1 -** Progreta mutant1; **PM2 -** Progreta mutant2; **S -** cv. Solara; **SM -** Solara mutante; **F -** cv. Frilene. **M** – DNA ladder

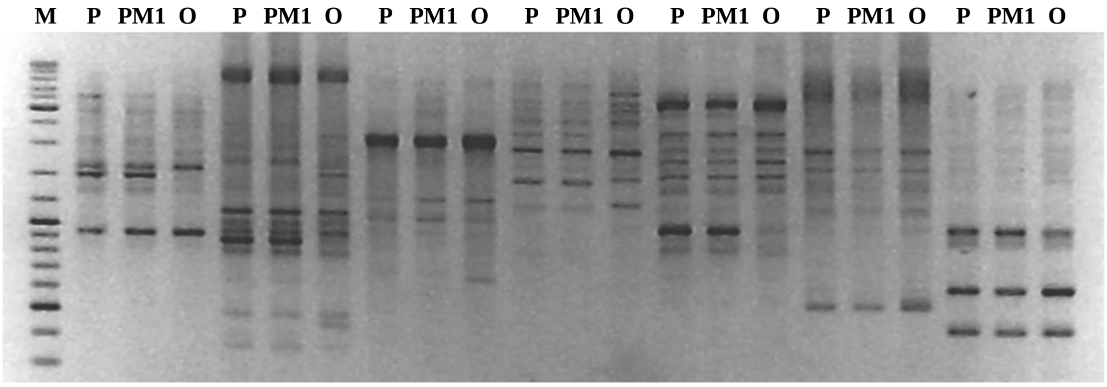


**Figure S2**. RAPD patterns of **P**- cv. Progreta, **PM1** _Progreta mutante 1 (SRP1), **O** – cv. Onward. **M** – DNA ladder

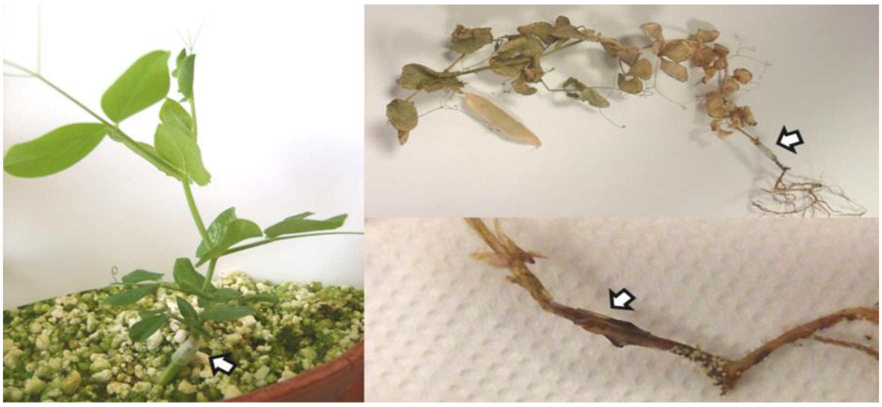

**Figure S3.** Plants of cv. Douce de Provence grafted on rogue *cv.* Progreta plants. (**Left**) Leaflets and stipula of the mature grafted plant exhibit the common non-rogue phenotype of *cv.* Douce de Provence. (**Right**) Senescent plant of cv. Douce de Provence grafted on rogue plant of *cv.* Progreta. Notice the regeneration of the plant tissue in the grafting region (white arrows).
